# Metabolic adaption of mucosal macrophages: Is metabolism a driver of persistence across tissues?

**DOI:** 10.1016/j.mucimm.2023.06.006

**Published:** 2023-10

**Authors:** Clare L. Bennett, Georgia Perona-Wright

**Affiliations:** 1 Department of Haematology, UCL Cancer Institute, University College London, London, UK; 2 School of Infection & Immunity, University of Glasgow, Glasgow, UK

## Abstract

Macrophages play essential roles in tissue homeostasis, defense, and repair. Their functions are highly tissue-specific, and when damage and inflammation stimulate repopulation by circulating monocytes, the incoming monocytes rapidly acquire the same, tissue-specific functions as the previous, resident macrophages. Several environmental factors are thought to guide the functional differentiation of recruited monocytes, including metabolic pressures imposed by the fuel sources available in each tissue. Here we discuss whether such a model of metabolic determinism can be applied to macrophage differentiation across barrier sites, from the lung to the skin. We suggest an alternative model, in which metabolic phenotype is a consequence of macrophage longevity rather than an early driver of tissue-specific adaption.

Macrophages are cells with multiple functions: they are phagocytes, engulfing and destroying microbial pathogens, clearing immune debris and apoptotic bodies^1^, ^2^. They are positioned at barrier sites throughout the body, offering first-line defense against infection. They also provide highly specialized, site-specific functions, from the mopping up of excess surfactant in the alveoli of the lungs, to iron metabolism in the spleen and synaptic pruning in the brain^3^. Tissue-specific macrophages are seeded by embryonic progenitors before birth, providing differentiated cells that are long-lived, capable of self-renewal, and educated by the local environment. Depending on the tissue anatomy, these resident macrophages can be replaced gradually by monocyte-derived cells that differentiate in the steady state to maintain local populations. More extreme replacement occurs in inflammation, infection, and/or immune pathology, which significantly deplete resident macrophage populations leading to an influx of monocyte-derived replacements^4^, ^5^.

Data suggest that the incoming cells acquire the phenotype and function of the resident cells that they replace once the inflammation has resolved, becoming almost (but not entirely) transcriptionally indistinguishable from the original tissue-dwelling cells^6^, ^7^, ^8^, ^9^, ^10^. This differentiation process suggests that a program of tissue-specific education instructs the incoming cells, determining their final function. A current hypothesis is that these tissue-specific signals are largely metabolic, with access to specific energy sources determining the possible routes of differentiation^11^, ^12^, ^13^. This model is well accepted in the lung, where the lipid-rich environment is associated with fatty acid oxidation and pro-repair or type 2-polarized macrophage function^14^. Here we discuss how this paradigm applies to other barrier tissues by focusing on the skin and gut, debating where and whether metabolism leads the tissue-specific differentiation process, or tissue adaption leads and metabolism follows.

Our review began as series of discussions between two colleagues, Clare Bennett, a Langerhans cell (LC) and macrophage expert working primarily in the skin, and Georgia Perona-Wright, a T cell and immunometabolism specialist focused on the gut and lung. Our discussion led us to consider the hypothesis that lipid metabolism does not confer macrophage specificity per se but is an adaptation for persistence and longevity that is shared across mucosal and other barrier sites. We have summarized our conversations here, exploring current thinking in the metabolic control of macrophage function.

## TISSUE SPECIFICATION OF MACROPHAGE FUNCTION

Tissue-resident macrophages are seeded by a relatively homogeneous population of embryonic progenitors that circulate before birth to populate nascent tissue niches^15^, ^16^, ^17^, ^18^. Here the core macrophage transcriptional program is overlaid by environmental signals present in each tissue leading to transcriptional and epigenetic changes that enforce differentiation of cells uniquely adapted to the needs of that tissue^18^, ^19^, ^20^, and equipped with the machinery needed for survival within that particular tissue environment. In some tissues such as the lung airway, the extrinsic and intrinsic signals that instruct this differentiation have been clearly delineated. For example, signaling by pulmonary transforming growth factor beta (TGFβ) and granulocyte-macrophage colony stimulating factor (GM-CSF) activates the transcription factors peroxisome proliferator-activated receptor gamma (PPARγ) and CCAAT enhancer binding protein beta (CEBPβ), which are required with early growth response 2 (EGR2) for alveolar macrophage (AM) differentiation and survival^21^, ^22^, ^23^, ^24^. Likewise, in serous cavities retinoic acid activates expression of GATA-6 and CEBPβ which together define tissue-specific macrophages at this site^25^, ^26^, ^27^. But while the core cytokines required for tissue macrophage specification have been identified in many tissues, there is considerable overlap and we still do not fully understand how niche factors combine to specify macrophage fate.

Fate-mapping studies in the mouse have revealed that over time, entry of bone marrow-derived monocytes leads to a certain ontogenic heterogeneity within tissue-resident macrophage populations^28^, ^29^. The extent to which embryo-derived macrophages persist in healthy tissues can be model-dependent, but it is clear that a spectrum exists across tissues: At one end, epidermal Langerhans Cells (LCs) and microglia in the brain become sealed in their compartments after birth^30^, ^31^, ^32^ and are maintained as quiescent, self-renewing, embryo-derived macrophage populations throughout life, without contribution from circulating cells. At the other extreme, the skin dermis and lamina propria of the gut are highly accessible to the bloodstream, and embryonic macrophages are rapidly replaced and maintained by a constitutive influx of blood monocytes throughout life^33^, ^34^. Ontogeny of tissue-specific macrophages may be critical in determining function, as has been discussed in detail elsewhere (e.g.^5^, ^35^). The ratio of different ontogenies in each tissue can also shift suddenly. Inflammation and destruction of resident macrophages due to viral infection or immune pathology, for example, initiates an influx of monocytes into inflamed sites to refill the empty niche^10^, ^36^. Monocyte-derived resident macrophages retain a bias toward a pro-inflammatory state and do not fully replicate the embryo-derived cells they replace until the local tissue environment has returned to steady-state homeostasis^10^, ^37^, ^38^. An interesting exception to this may be monocyte-derived LCs which we have shown rapidly differentiate within the inflamed epidermis^39^.

This transition of inflammatory monocytes into quiescent tissue macrophages involves a shift in energy requirement, supporting persistent, homeostatic function rather than the acute activity of an inflammatory response. So, to what extent do metabolic needs shape monocyte differentiation? Monocyte-derived AMs that differentiate in the lung after influenza-dependent destruction of resident embryo-derived macrophages do not initially exhibit lipid-dependent metabolism, but over time adopt this profile as they become more like the resident AMs they replace^10^. Does this transition illustrate that access to different metabolic fuels in their new location contributes to the functional programming of the incoming monocytes? What is known about the interplay between tissue specification of macrophage differentiation and metabolic restriction in the environment?

## METABOLIC REGULATION OF MACROPHAGE ACTIVATION

Immune cells that are actively participating in an immune response are radically different from cells at rest: they proliferate, alter surface proteins, and secrete cytokines and effector molecules. The biosynthesis involved in these changes imposes an acute energy demand on the cell, and early understanding of metabolic regulation argued that this demand was typically met by glycolysis^40^, ^41^. Glycolysis provides rapid ATP generation, and the increase in glucose uptake also drives the pentose phosphate pathway and the synthesis of lipid and amino acid precursors, needed for rapid cytokine and effector molecule production^42^. The increased glycolytic flux maintains essential redox balance (NAD+/NADH)^43^, ^44^, and the increased glutaminolysis associated with glycolysis drives the hexosamine biosynthetic pathway that produces essential substrates for the glycosylation of recently synthesized proteins^45^, an essential step for functional cytokine production. In contrast, resting cells use a slower but more efficient metabolism reliant on lipid consumption and oxidative catabolism, a decision thought to promote longevity and homeostatic quiescence^46^, ^47^. Resident memory T cells at barrier sites such as the skin are perhaps the poster child of quiescent immune cells, using lipids to fuel their persistence^48^.

Macrophages have also been described as existing in one of these two binary states: resting cells that are associated with slow and sustained fatty acid oxidation, and acutely activated cells characterized by intense glycolysis^49^. This glycolytic rush fuels the respiratory burst and the rapid production of reactive oxygen species (ROS), which is an essential part of macrophage pathogen clearance. Indeed, this metabolic switch is directly targeted by some pathogens as a means of escape (for example *Mycobacterium tuberculosis*^50^, ^51^). Activated macrophages are now known to exist in a wide variety of phenotypes, however, and many of these profiles have been associated with distinctive metabolic signatures (described in detail in recent reviews^11^, ^12^). *In vitro*, while *toll*-like receptor- and interferon-γ-stimulated macrophages are actively glycolytic, those with repair or resolution profiles such as macrophages activated in the presence of interleukin (IL)-4 or during helminth infection, are instead associated with mitochondrial metabolism and oxidative phosphorylation^52^, ^53^. *In vivo*, some elements of this dichotomy hold true: inflammatory macrophages show active glycolysis, and some helminth infections and other type II immune contexts have been associated with high oxidative phosphorylation in myeloid cells^12^.

**Fig. 1:**
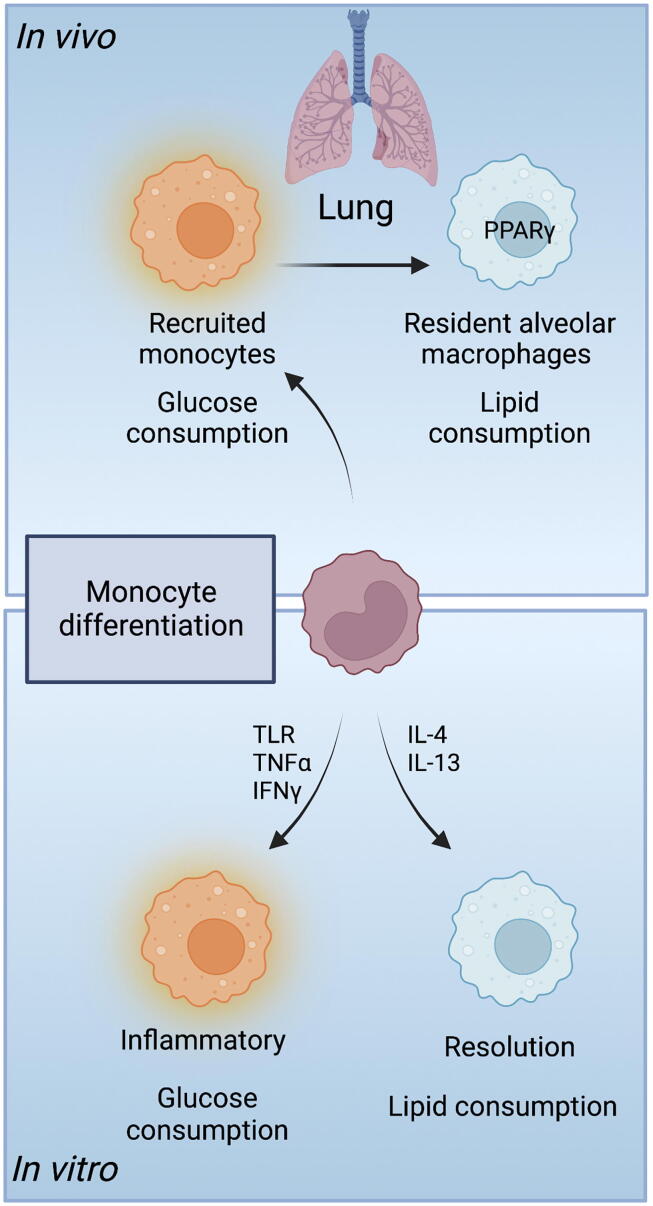
Current paradigms of macrophage metabolic adaptation. Schematic showing current models of metabolic influence on monocyte differentiation. (Bottom) *in vitro* studies have shown that inflammatory monocyte-derived macrophages use glucose as a dominant fuel source, while resolution-phenotype macrophages preferentially consume lipids. (Top) *in vivo*, in the lung, recruited monocyte-derived alveolar macrophages are initially glycolytic and adopt an inflammatory state. Differentiation to resident, quiescent, alveolar macrophages is linked to expression of the transcription factor PPARγ and a switch to lipid metabolism. IFN = interferon; IL = interleukin; PPARγ = peroxisome proliferator-activated receptor gamma; TLR = *toll*-like receptors; TNF = tumor necrosis factor.

Recent reports have shown, however, that *in vivo* tissue location and fuel availability are significant influences on the metabolic characteristics and functional potential of local macrophages. The lung is the best-studied example to date, perhaps in part due to the relative ease of isolating AMs; understanding of immune metabolism has often leaped forward with technological advances that capture improved in-depth, *in vivo,* and spatial information^54^, ^55^, ^56^ (Table 1). The airways are depleted of glucose, to deter microbial outgrowth, but are abundant in lipids such as surfactants. Location within this lipid-rich environment is thought to impose a lipid-dependent, highly oxidative metabolism on local AMs, restricting their inflammatory potential and promoting a resolution function^14^ (Fig. 1). Svedberg et al.^14^ directly compared the metabolic requirements of differentiated AMs to those of peritoneal macrophages, and demonstrated that residency within the alveolar space is associated both with the quiescent, resolving, lipid-metabolizing profile of PPARγ+ AMs, and coincident downregulation of glucose consumption. The link between PPARγ as the defining transcription factor of AMs and as a key regulator of lipid metabolism suggests a model in which the lipid-rich environment of the lung imposes a fatty acid dependency that activates PPARγ and initiates downstream signaling that enforces an airway macrophage phenotype. Consistent with this model, resident interstitial lung macrophages, which regulate a homeostatic environment within a more nutrient-diverse lung space, do not require PPARγ and remain more glycolytically active^51^, ^57^.

**Table 1:** Advances in methodology in immunometabolism. Understanding immune cell metabolism across models and in different tissue sites has progressed with significant developments in available methodologies. Classical biochemical approaches such as measuring metabolic flux with early Seahorse analysers relied on large numbers of cells, a factor which led to an initial dependence on in vitro cultures. Resident macrophages rapidly lose expression of genes that confer their tissue identity upon extraction from tissues, potentially confounding ex vivo analyses depending on the ease and speed with which the cells can be accessed and processed. The range of assays available in immunometabolism is rapidly expanding, particularly in single cell technologies based in flow cytometry and in systems-based approaches. New innovations in spatial transcriptomics and spatial metabolomics are beginning to provide detailed maps of metabolic environments, and future challenges include accessibility of these technologies and the addition of temporal information. Example publications are listed.

**Methodology**	**Tissue/Cells used**	**Pros**	**Cons**	
**In vitro cultures**				
*Extracellular flux analysis (eg. Seahorse analysis)*	Largely in vitro-generated macrophages (mouse bone marrow, human monocytes)	Direct functional measurement of metabolic activity	Requires abundant cells (100k+) that are stable in culture	109, 14, 59, 110
	Sorted primary cells		Impacted by culture conditions/medium used	
*Biochemical assays with addition of (labelled) substrates including stable isotope tracers*	Largely in vitro-generated macrophages (mouse bone marrow, human monocytes)	Direct functional measurement of metabolic activity	Requires abundant cells that are stable in culture	111, 112, 113
	Sorted primary cells		Impacted by culture conditions/medium used	
			Stable isotype labelling uses “heavy” elements	
**Ex vivo**				
***Flow cytometry***				
*Uptake of labelled or unlabelled substrates and dyes (e.g. BODIPY, Filipin III, mitotracker, glucose, amino acid metabolites)*	Tissue single cell suspensions	Measure of functional cellular activity at a specific point in time	Limited reagents to key pathways	114, 115
	Cell cultures	Can also be used for microscopy	Autofluorescence and toxicity can affect readouts	
*Met-flow, scMEP* (*single-cell metabolic regulome profiling) – detection of e.g. receptors, transporters and rate-limiting enzymes*	Tissue single cell suspensions	In depth metabolic phenotyping of tissue macrophages	Phenotypic rather than functional readout	116, 117
	Human peripheral blood mononuclear cells	Doesn’t require sorting of macrophages	May require extensive optimisation depending on the size of the flow cytometry panels	
		Practical for profiling multiple populations of tissue-resident macrophages and/or longitudinal analyses		
*SCENITH* (*Single Cell ENergetIc metabolism by profiling Translation inHibition)*	Tissue single cell suspensions	Captures the functional capacities of cells at the single cell level	Dependent on inhibition of pathways and measurement of protein synthesis as a readout for ATP production and metabolic activity; indirect measures	118, 119
	Human peripheral blood mononuclear cells	Doesn’t require sorting of macrophages	Not suitable for quiescent cells with low levels of protein synthesis	
		Amenable to low cell numbers (1000s)		
**“-omics”**				
*Proteomics (mass spectrometry)*	Sorted macrophage populations	Unbiased overview of proteins expressed by the cell	Capture of metabolism-associated proteins in small sample sizes may be obscured by abundant proteins such as cytoskeletal proteins	^120^
		Can be performed on low (100s) numbers of primary macrophages		
*Metabolomics (analysis of metabolites by mass-spectrometry)*	Whole tissues	Highly sensitive to small changes in metabolic pathways	Typically uses large numbers (millions) of cells, difficult to separate low metabolite levels from background when small numbers of cell are used. But single cell metabolomics technologies are being rapidly optimised	121, 122, 123
	Cultured cells	Untargeted approach allows discovery of global changes while targeted approaches are more sensitive to specific pathways of interest	Metabolite coverage strongly influenced by extraction techniques	
	Body fluids and cell supernatants	Can be performed at the single cell level	Large numbers of metabolites can make identifying and validating biologically meaningful differences challenging	
*Lipidomics (analysis of specific lipid species by mass-spectrometry or NMR)*	Whole tissues	Untargeted approach allows discovery of global changes while targeted approaches are more sensitive to specific pathways of interest	Vast diversity in lipid species makes analysis complex and/or requires targeted analysis	^124^
	Cultured cells	Compatible with tracer studies	Highly sensitive, so data can be noisy	
	Body fluids and cell supernatants			
*Single cell RNAseq with metabolic pathway analysis (eg. Compass, a flux balance algorithm that integrates metabolic states with single cell data)*	Sorted tissue-resident macrophages or total tissues for single cell analyses	Single cell analysis captures heterogeneity in cell states and gene expression at the point of cell isolation	May not give real-time information on key metabolic proteins – missing post-transcriptional regulation, and temporal differences between mRNA, protein abundance and metabolic flux	125, 55
			Analysis depends on quality and caveats associated with single cell RNA sequencing, e.g. depth of sequencing	
**In vivo**				
*Tracing labelled substrates (“fluxomics”)****(****e.g. using 18F-fluorodeoxyglucose with scanning techniques such as Positron Emission Tomography (PET)*	Whole mouse	In situ, real-time measurement of substrate usage by cells	PET requires limited substrates – glucose, glutamine metabolism	126, 127
	Human volunteers		May be limited by resolution of PET scans	
*Genetically modified mice*	Whole mouse	Defines a functional requirement for the gene/protein	Specificity depends on the Cre promoter; use of intersectional genetic approaches may improve specificity	59, 115, 128
*Spatial metabolomics with imaging mass spectrometry*	Tissue sections	Localisation of metabolites to defined tissue areas or potentially within cell organelles	Complicated sample preparation	129, 130
			Limitations in how material is separated compared to e.g. liquid chromatography-mass spectrometry (LC-MS) for conventional metabolomics; this increases overlap of signals, noise and variability

## MODELS OF METABOLIC INSTRUCTION

**Fig. 2:**
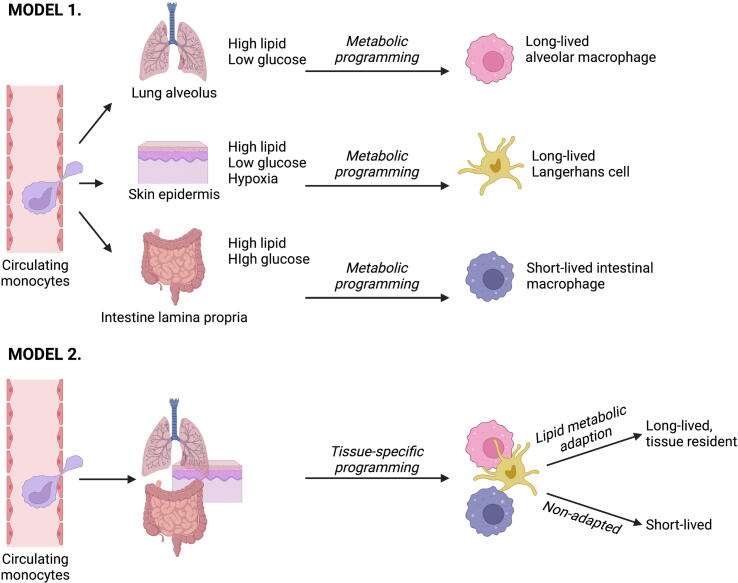
Alternative models of metabolic adaption in tissue macrophages. (Top) the idea of tissue-specific metabolic adaption suggests that the unique metabolic environments of different tissues impose distinct patterns of differentiation and function on incoming monocytes and resultant macrophages. (Bottom) an alternative model is that monocytes entering a tissue receive signals such as cytokines and growth factors that specify cell fate and function. When that cell fate is long-lived, the macrophages adapt for quiescence and persistence by adopting an efficient, often lipid-based metabolic profile. When the cell is instead destined for rapid function, short life and early death, the metabolic profile remains dominated by rapid, inefficient metabolic pathways such as glycolysis.

The idea that local fuel availability directs the metabolic and functional profile of tissue macrophages is often discussed in the context of the lung but may also hold true elsewhere. In the liver, for example, a high-fat diet results in differentiation of distinct lipid-associated macrophages due to activation of Trem2-dependent signaling^58^. These examples have established a concept of metabolism directing differentiation and function: access to specific fuels determines activation potential and cell identity. However, the current data would also be consistent with a model in which macrophage persistence and residency are associated with a switch to quiescent metabolism, irrespective of tissue-specific adaption (Fig. 2). Many mucosal and barrier sites are rich in lipids, and yet macrophage function is different and specific to each site. Sancho and colleagues have recently reported that the metabolic factor that most distinguishes macrophages at different sites is oxidative phosphorylation, a process that in T cells is associated with metabolic efficiency and long life^59^. Is it possible that the distinctive metabolic profiles seen in macrophages at different tissue sites reflect their life span and persistence, rather than being imposed by the local tissue environment?

## LC IN THE SKIN EPIDERMIS: LIPID METABOLISM AND CELL LONGEVITY

**Fig. 3:**
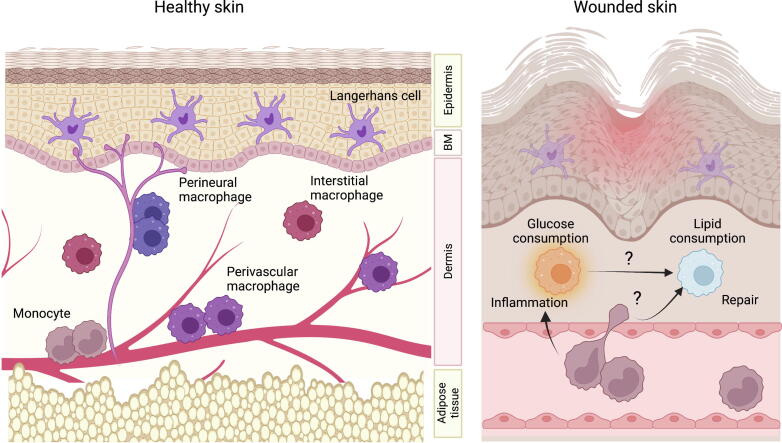
Skin macrophage populations in health and during wounding. (Left) healthy skin is populated by many resident macrophage populations. Langerhans cells reside in the lipid-rich epidermis, where in the steady state they are maintained without contribution from circulating monocytes. In the dermis, most but not all macrophages are maintained by recruited monocytes. Dermal macrophages reside in sub-tissular niches as interstitial cells or located around dermal blood vessels (shown in red) or peripheral nerves (shown in pale purple). The dermis is underlain by a layer of adipose tissue. (Right) after wounding, monocytes are recruited into the site of injury where they differentiate into macrophages. Early after wounding, monocyte-derived macrophages resemble inflammatory macrophages found at other sites, relying on glucose to fuel proliferation and anti-microbial functions. As the wound heals, gene expression in local macrophages becomes dominated by lipid-consumption and repair processes. Question marks indicate open questions about how metabolic and environmental signals intersect to co-ordinate the macrophage response to tissue repair, and whether inflammatory and reparative macrophages are seeded from the same monocytic precursors.

In the lung, airway macrophages are active in lipid-dependent, oxidative metabolism; are embedded in a lipid-rich environment; and are persistent, long-lived cells. Some insight into cause and effect in these associations can be gained from analysis of other lipid-rich sites. In the skin, for example, the epidermis is composed of layers of keratinocytes, constantly differentiating and moving away from the basal layer of the epidermis toward the outer edge of the skin. During this journey, the keratinocytes complete their lifespan, die, and are replaced^60^ (Fig. 3). These cells form the constantly replenished stratum corneum that protects our body from dehydration, infection, and physical insults^61^. Like the alveolar space, the epidermis is therefore very lipid-rich. Keratinocytes secrete several lipids, the composition of which changes as they migrate and differentiate away from the epidermal basement membrane; these include glycolipids and phospholipids that are broken down to produce free fatty acids, cholesterols that are progressively catabolized, and glucosylceramides that are converted into ceramides^62^. Production of lipids is fundamental to tissue function; the layers of lipid lamellae are said to provide the “mortar” sealing the keratinocyte building blocks together to maintain an impermeable physiological barrier, and to provide an immunological barrier containing anti-microbial fatty acids and a lipid-rich niche for commensal bacteria^62^. Skin microbiota provide essential amino acids and other metabolites that play important roles in regulating skin immunity, for example via the aryl hydrocarbon receptor. The epidermis also lacks blood vessels, making epidermal cells dependent on exposure to the air for oxygen, resulting in a relatively hypoxic environment^63^ and limiting nutrient concentrations including glucose at this site.

LCs in the skin, therefore, share several characteristics with AMs in the airways. They sit at the epithelial interface with the outside world. They persist as a self-renewing, remarkably long-lived population of epidermal macrophages, self-maintained throughout life without contribution from circulating monocytes. Evidence for this in people comes from recipients of hand allografts in which LCs from the donor are maintained in the skin of the transplanted limb for decades post-surgery^64^. LCs are seeded before birth from embryonic CX3CR1+ macrophage precursors and, driven by undefined signals, the population undergoes a burst of proliferation approximately 2 days after birth to fill the epidermal niche^65^. During the steady state LCs constitutively migrate at low levels to draining lymph nodes^66^, where they are thought to help maintain tolerance to innocuous skin antigens^67^. This migration is enhanced during infection or inflammatory disease, and, in this case, local proliferation of resident LCs is sufficient to refill gaps in the LC network^65^, ^68^.

Like AMs, LC identity at birth is partly conferred by TGFβ^69^, ^70^, but in the epidermis, LC cell fate is also dependent on IL-34^71^, ^72^ and bone morphogenetic protein 7 (BMP7)^73^, ^74^ which activate Id2 and Runx3^75^, ^76^. Once the LC network is established, IL-34 appears to be sufficient for long-term survival[71] while TGFβ is required for retention of LCs within the epidermis^77^, ^78^. The transcription factor dependence of LCs is specific to their location and different from that of AMs, despite the lipid-rich environment surrounding both populations. Immune cells in the skin do show a distinctive metabolic profile; differentiating T cells that enter the skin epidermis metabolize exogenous lipids to survive as long-lived resident memory cells, characterized by the upregulation of fatty acid binding proteins and the fatty acid translocase CD36^48^, ^79^. As observed for AMs, expression of PPARγ in epidermal T cells promotes uptake of fatty acids^48^. Transcriptomic sequencing of human LCs that have migrated out of epidermal sheets, or which have been released by trypsinization, suggests that lipid metabolism pathways dominate gene activity, including biosynthesis of unsaturated fatty acids, fatty acid metabolism, and amino acid metabolism^80^. Moreover, recent data suggest that LCs may cluster with AMs as high lipid- and cholesterol-processing macrophages that are dependent on oxidative phosphorylation^59^. Together these data suggest that, like AMs, LCs require lipid metabolism for maintenance as tissue-resident, quiescent cells, but the link between fuel use and differentiation is still uncertain. In addition, LC destruction by ultraviolet irradiation or pathogenic T cells activates recruitment of dermal monocytes across the basement membrane into the inflamed epidermis, wherein they differentiate into replacement LCs^36^, ^39^. This differentiation takes place despite the inflammation, keratinocyte damage, and likely dyslipidemia in this context, suggesting intrinsic programming of LC differentiation rather than a cell fate imposed by the metabolic environment. Interestingly, the accumulation of neutral lipids in LCs isolated from lesions in psoriatic patients has been linked to a downregulation in autophagy, a cellular mechanism used to break down intracellular debris and regulate lipid metabolism^81^. Loss of lipid catabolism in psoriatic LCs was associated with a switch to a more inflammatory phenotype that contributed to disease pathogenesis^81^, consistent with a model in which lipid metabolism defines long-lived, quiescent macrophages, and an inability to use this metabolic adaption imposes an acute, inflammatory, activated phenotype rather than controlling cell identity.

## SHORT-LIVED MACROPHAGES IN THE INTESTINE AND THE SKIN

The intestine is a site in which many fuels are readily abundant: it is rich in lipids associated with mucus and dietary fats, abundant in amino acids derived from food, and also well vascularized and hence glucose-rich too^82^. The presence of the microbiome also provides microbial metabolites that regulate intestinal immunity, including macrophage function^82^, ^83^. Most, but not all^84^, ^85^, macrophages in the healthy gut are constitutively replenished by circulating monocytes that differentiate over 5–6 days to become mature cells characterized by loss of proteins such as Ly6C, and instead high expression of the chemokine receptor CX3CR1^34^, ^86^, ^87^, ^88^. Mature lamina propria macrophages secrete chemokines that sustain monocyte recruitment^89^ and their life span is short, thought to be limited by a tonic state of low-grade inflammation stimulated by the combination of microflora and dietary challenge^90^.

The metabolism of intestinal macrophages is not yet completely described. Lamina propria macrophages in the large intestine are active in both glycolysis and oxidative phosphorylation^83^ and their metabolism increases further under germ-free or antibiotic conditions^91^, suggesting that microbial products such as butyrate are metabolically influential^92^. Small intestinal macrophages show lower rates of glycolysis, oxidative phosphorylation, and mTOR activity than those in the colon^83^. Interestingly, fatty acid uptake and lipid storage in intestinal macrophages has been associated only with a rare population of CD4+Tim4+ tissue residents in the colon^83^, ^85^. These cells are long-lived and self-renewing^85^, again associating lipid acquisition with macrophage persistence and longevity.

Another barrier location that is characterized by short macrophage life span and constitutive replenishment is the dermal layer of the skin^33^. As in the intestine, neurons and blood vessels provide some local niches in the dermis that instruct specialized macrophage differentiation and may protect longer-lived resident specialist macrophage sub-populations^93^, ^94^, ^95^, but the majority of macrophages in the dermal layer are continually replaced by circulating monocytes^29^, ^33^. The dermal layer is less lipid-rich than the epidermis and is intricately connected to the circulation, so it shares environmental and cellular similarities to the gut and oral mucosa. Contrary to human and murine LCs, the dermal monocyte/macrophage transcriptional program is dominated by genes associated with immune function rather than metabolic pathways^33^, ^80^, ^96^ . Differentiation of MerTK+CD64+ dermal macrophages was associated with upregulation of the fatty acid transporter gene *cd36* in the study by Tamoutounour et al.^33^, and dermal Trem2+ macrophages are enriched for pathways encoding cholesterol metabolisms and PPAR signaling. These cells have been linked to pathology in the context of dysregulated cutaneous lipids in acne development^97^. However, the metabolic changes associated with differentiation of dermal macrophage populations have not been investigated in detail. Given the constitutive turn-over of most dermal macrophages, one possibility is that switching to lipid metabolism pathways to provide long-term efficient fuel usage is not required. This would be consistent with the concept of the metabolism of tissue-specific macrophages as a late-stage adaptation enabling tissue persistence, rather than an early driver of differentiation.

The idea of tissue-specific adoption of slow burn metabolism as a feature of long-lived, resident macrophages, in contrast to the active metabolism of cells recently derived from infiltrating monocytes, is given extra complexity at barrier sites: the gut, skin, and lungs are all sites of multiple, frequent infections, and macrophage plasticity is an essential response to varied parasitic, viral, and bacterial challenges. Perhaps metabolic flexibility is needed in short-lived intestinal macrophage populations to preserve responsiveness to the rapidly changing environment? How are metabolic changes linked to cellular fates within changing tissue environments?

## PERTURBATION IN THE SKIN: METABOLIC RESPONSES TO WOUND HEALING

Clues to answering this question may come from our understanding of wound healing in the skin; here the adoption of different metabolic pathways by recruited immune cells is entwined with repair of tissue damage^41^, ^98^ (Fig. 3). Recent transcriptomic and functional analyses of macrophages at different time points post-wounding demonstrated that glycolysis/gluconeogenesis, Hif-1α, and pentose phosphate pathways dominate early macrophage gene programmes^99^, facilitating the macrophage microbicidal effector functions required to sterilize the wound. At subsequent time points, the expression of genes associated with fatty acid oxidation, lipid catabolism, and enhanced mitochondrial processes^99^ is associated with a switch to a pro-fibrotic resolution phenotype that orchestrates epidermal re-growth, stromal repair, and restoration of tissue homeostasis^98^. These studies highlight the coordinated acquisition of distinct metabolic pathways by recruited macrophages, with lipid uptake and catabolism associated with resolution. As in the intestine, where microbial metabolites can influence host cell behavior, in skin inflammation it has been suggested that metabolites produced during the initial inflammatory phase of the response activate differentiation of immune-suppressive macrophages^98^. For example, production of itaconate from citrate/aconitate is associated with suppression of inflammation^100^, ^101^. On the other hand, binding of IL-4Rα by IL-4 and IL-13 has a dominant immune-suppressive impact on macrophages during tissue repair^102^, and directly alters mitochondrial metabolism due to a switch from mitochondrial ROS production to oxidative phosphorylation^99^, suggesting that metabolic adaption may be a consequence, not a driver, of the switch from inflammatory to reparative macrophages. Similarly, activation of lipid metabolic pathways *per se* does not appear to be sufficient to regulate this switch: while excessive TNFα delays skin wound healing by suppressing differentiation of pro-resolution macrophages, this is due to the transcription factor activity of sterol response element binding protein 2, which switches on an inflammatory gene program in macrophages at the wound site, rather than due to suppression of lipid metabolism in these cells^103^.

Current models of skin wound healing infer homogeneity within recruited monocytes, such that all cells transition through both inflammatory and reparative states during the wound healing process; pseudo-time analysis of gene expression programs in adoptively transferred macrophages supports this differentiation trajectory^104^, perhaps as part of an evolutionarily conserved response to protect the tissue^41^. But significant heterogeneity exists within macrophages isolated from the skin wound site^99^, and an alternative hypothesis is that highly activated glycolytic monocyte-derived macrophages die and are replaced by intrinsically different cells that enter the wound later in the healing process to become the resident (lipid-dependent) macrophage population^105^. In the lung, inflammatory AMs activated by influenza virus infection that express high levels of Hif-1a are more likely to die, while the Hif-1a^low^ less inflammatory cells proliferate and preferentially expand to repair the damaged lung^106^. Rodero et al.^107^ observed two waves of monocytes entering wounded skin by intravital imaging; Lyc6^high^ inflammatory monocytes were rapidly recruited into the wound, while Ly6C^low^CX3CR1^high^ monocytes arrived later during the repair process. While the fate of these cells has not been determined, it is tempting to speculate that they may represent independent precursors of glycolytic pro-inflammatory and lipid metabolizing resolution macrophages required during wound healing. More detailed barcoding and fate-mapping studies are needed to resolve this question and to determine how findings in the skin apply to other tissue contexts.

## CURRENT QUESTIONS

The adaptions of monocyte-derived cells during wound healing in the skin clearly show that metabolism and function are closely linked, but cause and effect are hard to distinguish. It may even be semantic to separate the two. Wculek et al.^59^ have recently identified lipid metabolism and oxidative phosphorylation as defining metabolic features that distinguish different tissue macrophage populations. Their work, together with that of Gao et al.^108^, demonstrates that macrophages in tissues with high lipid content, such as the lung alveolar space, are more dependent on oxidative phosphorylation to generate energy and thereby more sensitive to interference with this pathway^59^ than macrophages in sites with lower lipid content. This is consistent with a model in which, under homeostatic conditions, local availability of lipids drives dependence on metabolic pathways that in turn enable tissue macrophage survival across tissues. As we begin to understand more about how metabolism and differentiation interact during homeostasis, we can begin to address the extent to which metabolic plasticity is retained within differentiated macrophages during infection and disease. We also currently understand little about how energetic requirements drive macrophage differentiation in the gut and the extent to which it is similar or different to the dermis. An interesting point is that the use of lipid catabolism pathways by resolving macrophages in the skin mirrors tissue repair mechanisms in the gut after helminth infection^98^. This raises the question of how gut macrophages, or those at other sites, adapt to competition for fuels or the changing nutrient environment imposed by the presence of pathogens or shifts in microbiota imposed by chronic infections. Barrier sites frequently encounter concurrent and successive infections so the programming of tissue-resident macrophages, both functional and metabolic, requires ongoing flexibility.

## SUMMARY

Metabolism is a key part of tissue differentiation. Metabolism and function are inherently linked, reflecting local signals, fuel availability, and environmental changes during and after inflammation. We argue that the current AM paradigm represents a simplified but useful model for understanding how energy usage intersects with macrophage differentiation and function, and our conversation has suggested that rather than instructing cell fate, macrophage metabolism might instead be an outcome of functional adaption. The interaction between transcription factor expression and metabolic phenotype may be different in different tissues, and the skin offers new insight into metabolic adaption of tissue-specific macrophages.

## AUTHOR CONTRIBUTIONS

CB and GPW drafted and edited the manuscript and designed all figures. Both authors approved the final manuscript.

## DECLARATIONS OF COMPETING INTEREST

The authors declare no competing interests. GPW supervises a PhD student funded in part by Sitryx Therapeutics.

## FUNDING

CB is funded by the Biotechnology and Biological Sciences Research Council, grant BB/T005246/1, and GPW by the Medical Research Council, grant MR/S009779/1.

## References

[1] Wynn T.A., Chawla A., Pollard J.W. (2013). Macrophage biology in development, homeostasis and disease. Nature.

[2] Underhill D.M., Gordon S., Imhof B.A., Nunez G., Bousso P. (2016). Élie Metchnikoff (1845–1916): celebrating 100 years of cellular immunology and beyond. Nat. Rev. Immunol..

[3] Park M.D., Silvin A., Ginhoux F., Merad M. (2022). Macrophages in health and disease. Cell.

[4] Ginhoux F., Guilliams M. (2016). Tissue-resident macrophage ontogeny and homeostasis. Immunity.

[5] Blériot C., Chakarov S., Ginhoux F. (2020). Determinants of resident tissue macrophage identity and function. Immunity.

[6] Scott C.L. (2016). Bone marrow-derived monocytes give rise to self-renewing and fully differentiated Kupffer cells. Nat. Commun..

[7] van de Laar L. (2016). Yolk sac macrophages, fetal liver, and adult monocytes can colonize an empty niche and develop into functional tissue-resident macrophages. Immunity.

[8] Bennett F.C. (2018). A combination of ontogeny and CNS environment establishes microglial identity. Neuron.

[9] Bonnardel J. (2019). Stellate cells, hepatocytes, and endothelial cells imprint the Kupffer cell identity on monocytes colonizing the liver macrophage niche. Immunity.

[10] Aegerter H. (2020). Influenza-induced monocyte-derived alveolar macrophages confer prolonged antibacterial protection. Nat. Immunol..

[11] Caputa G., Castoldi A., Pearce E.J. (2019). Metabolic adaptations of tissue-resident immune cells. Nat. Immunol..

[12] Wculek S.K., Dunphy G., Heras-Murillo I., Mastrangelo A., Sancho D. (2022). Metabolism of tissue macrophages in homeostasis and pathology. Cell. Mol. Immunol..

[13] Jung J., Zeng H., Horng T. (2019). Metabolism as a guiding force for immunity. Nat. Cell Biol..

[14] Svedberg F.R. (2019). The lung environment controls alveolar macrophage metabolism and responsiveness in type 2 inflammation. Nat. Immunol..

[15] Gomez Perdiguero E. (2015). Tissue-resident macrophages originate from yolk-sac-derived erythro-myeloid progenitors. Nature.

[16] Schulz C. (2012). A lineage of myeloid cells independent of Myb and hematopoietic stem cells. Science.

[17] Hoeffel G. (2015). C-Myb(+) erythro-myeloid progenitor-derived fetal monocytes give rise to adult tissue-resident macrophages. Immunity.

[18] Mass E. (2016). Specification of tissue-resident macrophages during organogenesis. Science.

[19] Gosselin D. (2014). Environment drives selection and function of enhancers controlling tissue-specific macrophage identities. Cell.

[20] Lavin Y. (2014). Tissue-resident macrophage enhancer landscapes are shaped by the local microenvironment. Cell.

[21] Schneider C. (2014). Induction of the nuclear receptor PPAR-gamma by the cytokine GM-CSF is critical for the differentiation of fetal monocytes into alveolar macrophages. Nat. Immunol..

[22] Yu X. (2017). The cytokine TGF-beta promotes the development and homeostasis of alveolar macrophages. Immunity.

[23] Bain C.C., MacDonald A.S. (2022). The impact of the lung environment on macrophage development, activation and function: diversity in the face of adversity. Mucosal Immunol..

[24] McCowan J. (2021). The transcription factor EGR2 is indispensable for tissue-specific imprinting of alveolar macrophages in health and tissue repair. Sci. Immunol..

[25] Gautier E.L. (2014). Gata6 regulates aspartoacylase expression in resident peritoneal macrophages and controls their survival. J. Exp. Med..

[26] Okabe Y., Medzhitov R. (2014). Tissue-specific signals control reversible program of localization and functional polarization of macrophages. Cell.

[27] Bain C.C. (2016). Long-lived self-renewing bone marrow-derived macrophages displace embryo-derived cells to inhabit adult serous cavities. Nat. Commun..

[28] Yona S. (2013). Fate mapping reveals origins and dynamics of monocytes and tissue macrophages under homeostasis. Immunity.

[29] Liu Z. (2019). Fate mapping via Ms4a3-expression history traces monocyte-derived cells. Cell.

[30] Merad M. (2002). Langerhans cells renew in the skin throughout life under steady-state conditions. Nat. Immunol..

[31] Ginhoux F. (2010). Fate mapping analysis reveals that adult microglia derive from primitive macrophages. Science.

[32] Hoeffel G. (2012). Adult Langerhans cells derive predominantly from embryonic fetal liver monocytes with a minor contribution of yolk sac-derived macrophages. J. Exp. Med..

[33] Tamoutounour S. (2013). Origins and functional specialization of macrophages and of conventional and monocyte-derived dendritic cells in mouse skin. Immunity.

[34] Bain C.C. (2014). Constant replenishment from circulating monocytes maintains the macrophage pool in the intestine of adult mice. Nat. Immunol..

[35] Jenkins S.J., Allen J.E. (2021). The expanding world of tissue-resident macrophages. Eur. J. Immunol..

[36] Ginhoux F. (2006). Langerhans cells arise from monocytes in vivo. Nat. Immunol..

[37] Epelman S. (2014). Embryonic and adult-derived resident cardiac macrophages are maintained through distinct mechanisms at steady state and during inflammation. Immunity.

[38] Cronk J.C. (2018). Peripherally derived macrophages can engraft the brain independent of irradiation and maintain an identity distinct from microglia. J. Exp. Med..

[39] Ferrer I.R. (2019). A wave of monocytes is recruited to replenish the long-term Langerhans cell network after immune injury. Sci. Immunol..

[40] O'Neill L.A., Kishton R.J., Rathmell J. (2016). A guide to immunometabolism for immunologists. Nat. Rev. Immunol..

[41] Wang A., Luan H.H., Medzhitov R. (2019). An evolutionary perspective on immunometabolism. Science.

[42] Donnelly R.P., Finlay D.K. (2015). Glucose, glycolysis and lymphocyte responses. Mol. Immunol..

[43] Anastasiou D. (2011). Inhibition of pyruvate kinase M2 by reactive oxygen species contributes to cellular antioxidant responses. Science.

[44] the metabolic requirements of cell proliferation (2009). Vander Heiden, M.G., Cantley, L.C. & Thompson, C.B. Understanding the Warburg effect. Science.

[45] Swamy M. (2016). Glucose and glutamine fuel protein O-GlcNAcylation to control T cell self-renewal and malignancy. Nat. Immunol..

[46] Lam W.Y. (2016). Mitochondrial pyruvate import promotes long-term survival of antibody-secreting plasma cells. Immunity.

[47] van der Windt G.J. (2012). Mitochondrial respiratory capacity is a critical regulator of CD8+ T cell memory development. Immunity.

[48] Pan Y. (2017). Survival of tissue-resident memory T cells requires exogenous lipid uptake and metabolism. Nature.

[49] Newsholme P., Curi R., Gordon S., Newsholme E.A. (1986). Metabolism of glucose, glutamine, long-chain fatty acids and ketone bodies by murine macrophages. Biochem. J..

[50] Hackett E.E. (2020). Mycobacterium tuberculosis limits host glycolysis and IL-1β by restriction of PFK-M via MicroRNA-21. Cell Rep..

[51] Huang L., Nazarova E.V., Tan S., Liu Y., Russell D.G. (2018). Growth of Mycobacterium tuberculosis in vivo segregates with host macrophage metabolism and ontogeny. J. Exp. Med..

[52] Jha A.K. (2015). Network integration of parallel metabolic and transcriptional data reveals metabolic modules that regulate macrophage polarization. Immunity.

[53] Quinteros S.L. (2023). The helminth derived peptide FhHDM-1 redirects macrophage metabolism towards glutaminolysis to regulate the pro-inflammatory response. Front. Immunol..

[54] Tans R. (2021). Spatially resolved immunometabolism to understand infectious disease progression. Front. Microbiol..

[55] Purohit V., Wagner A., Yosef N., Kuchroo V.K. (2022). Systems-based approaches to study immunometabolism. Cell. Mol. Immunol..

[56] Artyomov M.N., Van den Bossche J. (2020). Immunometabolism in the single-cell era. Cell Metab..

[57] Zhou B. (2020). The angiocrine Rspondin3 instructs interstitial macrophage transition via metabolic-epigenetic reprogramming and resolves inflammatory injury. Nat. Immunol..

[58] Jaitin D.A. (2019). Lipid-associated macrophages control metabolic homeostasis in a Trem2-dependent manner. Cell.

[59] Wculek S.K. (2023). Oxidative phosphorylation selectively orchestrates tissue macrophage homeostasis. Immunity.

[60] Klicznik M.M., Szenes-Nagy A.B., Campbell D.J., Gratz I.K. (2018). Taking the lead - how keratinocytes orchestrate skin T cell immunity. Immunol. Lett..

[61] Kabashima K., Honda T., Ginhoux F., Egawa G. (2019). The immunological anatomy of the skin. Nat. Rev. Immunol..

[62] Vietri Rudan M., Watt F.M. (2021). Mammalian epidermis: a compendium of lipid functionality. Front. Physiol..

[63] Rezvani H.R. (2011). HIF-1alpha in epidermis: oxygen sensing, cutaneous angiogenesis, cancer, and non-cancer disorders. J. Invest. Dermatol..

[64] Kanitakis J., Morelon E., Petruzzo P., Badet L., Dubernard J.M. (2011). Self-renewal capacity of human epidermal Langerhans cells: observations made on a composite tissue allograft. Exp. Dermatol.

[65] Chorro L. (2009). Langerhans cell (LC) proliferation mediates neonatal development, homeostasis, and inflammation-associated expansion of the epidermal LC network. J. Exp. Med..

[66] Kissenpfennig A. (2005). Dynamics and function of Langerhans cells in vivo: dermal dendritic cells colonize lymph node areas distinct from slower migrating Langerhans cells. Immunity.

[67] Seneschal J., Clark R.A., Gehad A., Baecher-Allan C.M., Kupper T.S. (2012). Human epidermal Langerhans cells maintain immune homeostasis in skin by activating skin resident regulatory T cells. Immunity.

[68] Ghigo C. (2013). Multicolor fate mapping of Langerhans cell homeostasis. J. Exp. Med..

[69] Kaplan D.H. (2007). Autocrine/paracrine TGFbeta1 is required for the development of epidermal Langerhans cells. J. Exp. Med..

[70] Kel J.M., Girard-Madoux M.J., Reizis B., Clausen B.E. (2010). TGF-beta is required to maintain the pool of immature Langerhans cells in the epidermis. J. Immunol..

[71] Greter M. (2012). Stroma-derived Interleukin-34 controls the development and maintenance of Langerhans cells and the maintenance of microglia. Immunity.

[72] Wang Y. (2012). IL-34 is a tissue-restricted ligand of CSF1R required for the development of Langerhans cells and microglia. Nat. Immunol..

[73] Tenno M. (2017). Cbfbeta2 deficiency preserves Langerhans cell precursors by lack of selective TGFbeta receptor signaling. J. Exp. Med..

[74] Yasmin N. (2013). Identification of bone morphogenetic protein 7 (BMP7) as an instructive factor for human epidermal Langerhans cell differentiation. J. Exp. Med..

[75] Chopin M. (2013). Langerhans cells are generated by two distinct PU.1-dependent transcriptional networks. J. Exp. Med..

[76] Seré K. (2012). Two distinct types of Langerhans cells populate the skin during steady state and inflammation. Immunity.

[77] Bobr A. (2012). Autocrine/paracrine TGF-beta1 inhibits Langerhans cell migration. Proc. Natl Acad. Sci. U. S. A..

[78] Mohammed J. (2016). Stromal cells control the epithelial residence of DCs and memory T cells by regulated activation of TGF-β. Nat. Immunol..

[79] Pan Y., Kupper T.S. (2018). Metabolic reprogramming and longevity of tissue-resident memory T cells. Front. Immunol..

[80] Polak M.E. (2014). Distinct molecular signature of human skin Langerhans cells denotes critical differences in cutaneous dendritic cell immune regulation. J. Invest. Dermatol..

[81] Zhang X. (2022). Abnormal lipid metabolism in epidermal Langerhans cells mediates psoriasis-like dermatitis. JCI Insight.

[82] Gu B.H., Kim M., Yun C.H. (2021). Regulation of gastrointestinal immunity by metabolites. Nutrients.

[83] Scott N.A., Mann E.R. (2020). Regulation of mononuclear phagocyte function by the microbiota at mucosal sites. Immunology.

[84] De Schepper S. (2019). Self-maintaining gut macrophages are essential for intestinal homeostasis. Cell.

[85] Shaw T.N. (2018). Tissue-resident macrophages in the intestine are long lived and defined by Tim-4 and CD4 expression. J. Exp. Med..

[86] Grainger J.R., Konkel J.E., Zangerle-Murray T., Shaw T.N. (2017). Macrophages in gastrointestinal homeostasis and inflammation. Pflugers Arch..

[87] Chiaranunt P., Tai S.L., Ngai L., Mortha A. (2021). Beyond immunity: underappreciated functions of intestinal macrophages. Front. Immunol..

[88] Denning T.L. (2011). Functional specializations of intestinal dendritic cell and macrophage subsets that control Th17 and regulatory T cell responses are dependent on the T cell/APC ratio, source of mouse strain, and regional localization. J. Immunol..

[89] Schridde A. (2017). Tissue-specific differentiation of colonic macrophages requires TGFbeta receptor-mediated signaling. Mucosal Immunol..

[90] Viola M.F., Boeckxstaens G. (2021). Niche-specific functional heterogeneity of intestinal resident macrophages. Gut.

[91] Scott N.A. (2018). Antibiotics induce sustained dysregulation of intestinal T cell immunity by perturbing macrophage homeostasis. Sci. Transl. Med..

[92] Schulthess J. (2019). The short chain fatty acid butyrate imprints an antimicrobial program in macrophages. Immunity.

[93] Haniffa M. (2009). Differential rates of replacement of human dermal dendritic cells and macrophages during hematopoietic stem cell transplantation. J. Exp. Med..

[94] Chakarov S. (2019). Two distinct interstitial macrophage populations coexist across tissues in specific subtissular niches. Science.

[95] Kolter J. (2019). A subset of skin macrophages contributes to the surveillance and regeneration of local nerves. Immunity.

[96] West H.C. (2022). Loss of T cell tolerance in the skin following immunopathology is linked to failed restoration of the dermal niche by recruited macrophages. Cell Rep..

[97] Do T.H. (2022). TREM2 macrophages induced by human lipids drive inflammation in acne lesions. Sci. Immunol..

[98] Eming S.A., Murray P.J., Pearce E.J. (2021). Metabolic orchestration of the wound healing response. Cell Metab..

[99] Willenborg S. (2021). Mitochondrial metabolism coordinates stage-specific repair processes in macrophages during wound healing. Cell Metab..

[100] Bambouskova M. (2018). Electrophilic properties of itaconate and derivatives regulate the IκBζ-ATF3 inflammatory axis. Nature.

[101] Lampropoulou V. (2016). Itaconate links inhibition of succinate dehydrogenase with macrophage metabolic remodeling and regulation of inflammation. Cell Metab..

[102] Allen J.E. (2023). IL-4 and IL-13: regulators and effectors of wound repair. Annu. Rev. Immunol..

[103] Kusnadi A. (2019). The cytokine TNF promotes transcription factor SREBP activity and binding to inflammatory genes to activate macrophages and limit tissue repair. Immunity.

[104] Sanin D.E. (2022). A common framework of monocyte-derived macrophage activation. Sci. Immunol..

[105] Minutti C.M., Knipper J.A., Allen J.E., Zaiss D.M. (2017). Tissue-specific contribution of macrophages to wound healing. Semin. Cell Dev. Biol..

[106] Zhu B. (2021). Uncoupling of macrophage inflammation from self-renewal modulates host recovery from respiratory viral infection. Immunity.

[107] Rodero M.P. (2014). In vivo imaging reveals a pioneer wave of monocyte recruitment into mouse skin wounds. PLoS One.

[108] Gao X. (2022). TFAM-dependent mitochondrial metabolism is required for alveolar macrophage maintenance and homeostasis. J. Immunol..

[109] Davies L.C. (2017). Peritoneal tissue-resident macrophages are metabolically poised to engage microbes using tissue-niche fuels. Nat. Commun..

[110] Huang S.C. (2016). Metabolic reprogramming mediated by the mTORC2-IRF4 signaling axis is essential for macrophage alternative activation. Immunity.

[111] Castoldi A. (2020). Triacylglycerol synthesis enhances macrophage inflammatory function. Nat. Commun..

[112] Geeraerts X. (2021). Macrophages are metabolically heterogeneous within the tumor microenvironment. Cell Rep..

[113] Violante S., Berisa M., Thomas T.H., Cross J.R. (2019). Stable isotope tracers for metabolic pathway analysis. Methods Mol. Biol..

[114] Sinclair L.V., Neyens D., Ramsay G., Taylor P.M., Cantrell D.A. (2018). Single cell analysis of kynurenine and System L amino acid transport in T cells. Nat. Commun..

[115] Di Conza G. (2021). Tumor-induced reshuffling of lipid composition on the endoplasmic reticulum membrane sustains macrophage survival and pro-tumorigenic activity. Nat. Immunol..

[116] Hartmann F.J. (2021). Single-cell metabolic profiling of human cytotoxic T cells. Nat. Biotechnol..

[117] Ahl P.J. (2020). Met-Flow, a strategy for single-cell metabolic analysis highlights dynamic changes in immune subpopulations. Commun. Biol..

[118] Adamik J. (2022). Distinct metabolic states guide maturation of inflammatory and tolerogenic dendritic cells. Nat. Commun..

[119] Argüello R.J. (2020). SCENITH: a flow cytometry-based method to functionally profile energy metabolism with single-cell resolution. Cell Metab..

[120] Qie J. (2022). Integrated proteomic and transcriptomic landscape of macrophages in mouse tissues. Nat. Commun..

[121] Vrieling F. (2020). Analyzing the impact of Mycobacterium tuberculosis infection on primary human macrophages by combined exploratory and targeted metabolomics. Sci. Rep..

[122] Johnson C.H., Ivanisevic J., Siuzdak G. (2016). Metabolomics: beyond biomarkers and towards mechanisms. Nat. Rev. Mol. Cell Biol..

[123] Lanekoff I., Sharma V.V., Marques C. (2022). Single-cell metabolomics: where are we and where are we going?. Curr. Opin. Biotechnol..

[124] Morgan P.K. (2021). Macrophage polarization state affects lipid composition and the channeling of exogenous fatty acids into endogenous lipid pools. J. Biol. Chem..

[125] Wagner A. (2021). Metabolic modeling of single Th17 cells reveals regulators of autoimmunity. Cell.

[126] Reinfeld B.I. (2021). Cell-programmed nutrient partitioning in the tumour microenvironment. Nature.

[127] Sheldon R.D., Ma E.H., DeCamp L.M., Williams K.S., Jones R.G. (2021). Interrogating in vivo T-cell metabolism in mice using stable isotope labeling metabolomics and rapid cell sorting. Nat. Protoc..

[128] Kim J.S. (2021). A binary Cre transgenic approach dissects microglia and CNS border-associated macrophages. Immunity.

[129] Alexandrov T. (2020). Spatial metabolomics and imaging mass spectrometry in the age of artificial intelligence. Annu. Rev. Biomed. Data Sci..

[130] Ganesh S. (2021). Spatially resolved 3D metabolomic profiling in tissues. Sci. Adv..

